# High CTLA-4 expression correlates with poor prognosis in thymoma patients

**DOI:** 10.18632/oncotarget.24645

**Published:** 2018-03-30

**Authors:** Giorgio Santoni, Consuelo Amantini, Maria Beatrice Morelli, Daniele Tomassoni, Matteo Santoni, Oliviero Marinelli, Massimo Nabissi, Claudio Cardinali, Vittorio Paolucci, Mariangela Torniai, Silvia Rinaldi, Francesca Morgese, Giovanni Bernardini, Rossana Berardi

**Affiliations:** ^1^ School of Pharmacy, University of Camerino, Camerino, Italy; ^2^ School of Biosciences and Veterinary Medicine, University of Camerino, Camerino, Italy; ^3^ Department of Molecular Medicine, Sapienza University of Rome, Rome, Italy; ^4^ Medical Oncology Unit, Università Politecnica delle Marche, Azienda Ospedaliero-Universitaria Ospedali Riuniti Umberto I-Lancisi, Salesi di Ancona, Italy; ^5^ I.N.M. Neuromed, Pozzilli, Isernia (IS), Italy

**Keywords:** cytotoxic T lymphocyte antigen 4 (CTLA-4), thymoma, overall survival (OS), tumor-infiltrating leukocytes (TILs)

## Abstract

Thymomas, tumors that arise from epithelial cells of the thymus gland, are the most common neoplasms of the anterior mediastinum, with an incidence rate of approximately 2.5 per million/year. Cytotoxic T Lymphocyte Antigen 4 (CTLA-4 or CD152) exerts inhibitory activity on T cells, and since its oncogenic role in the progression of different types of tumors, it has emerged as a potential therapeutic target in cancer patients.

In this study, we assessed the expression of CTLA-4 both at mRNA and protein levels in paraffin embedded-tissues from patients with thymomas. Furthermore, we evaluated the relationship between CTLA-4 expression and the clinical-pathologic characteristics and prognosis in patients with thymomas. Sixty-eight patients with median age corresponding to 62 years were included in this analysis. Thymomas were classified accordingly to the WHO and Masaoka-Koga for histochemical analysis and for prognostic significance.

A statistical difference was found between CTLA-4 mRNA levels in human normal thymus compared with thymoma specimens. CTLA-4 expression was statistically found to progressively increase in A, B1, B2, AB and it was maximal in B3 thymomas. According to Masaoka-Koga pathological classification, CTLA-4 expression was lower in I, IIA and IIB, and higher in invasive III and IV stages. By confocal microscopy analysis we identified the expression of CTLA-4 both in tumor cells and in CD45^+^ tumor-infiltrating leukocytes, mainly in B3 and AB thymomas.

Finally, CTLA-4 overexpression significantly correlates with reduced overall survival in thymoma patients and in atypical thymoma subgroup, suggesting that it represents a negative prognostic factor.

## INTRODUCTION

Thymomas, tumors that arise from epithelial cells of the thymus gland, are the most common neoplasms of the anterior mediastinum, with an incidence rate of approximately 2.5 per million/year [1]. Thymomas are rare in patients under 25 years of age and show a wide age distribution, with a mean of incidence around 50–60 years of age, without a major sex predilection [1]. Although thymomas are, in general, indolent neoplasms, they are considered as malignant, irrespective to different subtypes.

Thymic neoplasms are divided according to the WHO classification [1], which is based on the premise that thymoma cells can belong to two histologic types: spindle/oval (designated as type “A”) or round/epithelioid (designated as type “B”). The type B were additionally subclassified based on the proportional increase in infiltrating lymphocytes and emergence of atypia of the neoplastic epithelial cells into B1, B2 and B3 subtypes. Finally a C category (thymic carcinoma) displaying cytological features of malignancy, including marked atypia, nuclear pleomorphism and high mitotic activity is accounted [2–5]. The Masaoka-Koga stage classification distinguished thymic malignancies in not-invasive I and IIA and invasive IIB, IIIA, IIIB, IVA and IVB stages [6]. Furtherly, accordingly to Moran and Suster classification, a more simplified approach in classifying the thymic epithelial neoplasms based on the histologic grading in well-differentiated (A, AB, B1 and B2, typical thymoma), moderately differentiated (B3, atypical thymoma) and poorly differentiated (C, thymic carcinoma), has been proposed [6–10]. Type AB thymoma is not considered a mixed tumor of type A and type B thymomas, but a distinct type of thymoma derived from a mixture of type A- and type B-like component positive for E-cadherin and negative for vimentin or a mixture of type B-like components and metaplastic mesenchymal components, positive for vimentin and negative for E-cadherin [11]. Once a tumor has been assigned to a differentiation category of thymic epithelial neoplasms, reliable prognostication can be determined by clinical and pathological Masaoka-Koga staging of the lesions. However, in several cases, histologic classification, according to WHO and/or pathological Masaoka-Koga stage classifications, cannot be completely correlated with clinical outcome [12], so the need to identify new prognostic biomarkers. In addition, an association between myasthenia gravis (MG), a neuromuscular disorder characterized by a defective transmission of nerve impulses to muscles, and thymoma has been reported [13]. About forty percent of the thymoma patients had associated MG, and preoperative absence of MG has been considered an independent predictor of poorer overall survival (OS) [12]. This disease is caused by an autoimmune reaction against components of the neuromuscular junction on the post-synaptic membrane of the striated skeletal muscles [13] and it is present at first diagnosis in up to a third of thymoma patients [14].

Cytotoxic T lymphocyte antigen-4 (CTLA-4, CD152) is an immune checkpoint molecule and a CD28 homologue that binds the ligands B7-1 (CD80) and B7-2 (CD86) [15]. Human CTLA-4 is present as a full-length membrane-bound receptor and as a secreted soluble molecule [16, 17]. Both the two isoforms reduce T cell activation by forming a negative feedback to maintain immune self-tolerance and homeostasis. CTLA-4 outcompetes CD28 for B7 ligands, attenuating the T cell response through the inhibition of IL-2 and blockage of cell cycle progression [18]. CTLA-4 is constitutively expressed at low levels on the surface of naïve, effector T (Teff) and regulatory T cells (Treg). The expression of CTLA-4 on Tregs reduces the levels of B7 ligands on antigen presenting cells. CTLA-4 has been also implicated in immune dysregulation of B cell chronic lymphocytic leukemia [19] and non Hodking's lymphoma [20].

The CTLA-4 molecule is expressed on normal non-lymphoid cells including placental fibroblasts [21], cultured muscle cells [22], monocytes [23] and mature dendritic cells [24]. In addition, it has been demonstrated that CTLA-4 is constitutively expressed not only in leukemia cells [25], but also in several types of tumor-derived cell lines including breast, colon, renal, lung, ovarian, uterine, bladder carcinoma, osteo/rabdomyosarcoma, neuroblastoma and melanoma [26] and in cancer tissues, such as osteosarcomas [26], non-small cell lung [27] and breast [28], nasopharyngeal [29], gastric [30] and esophageal carcinomas [31] and mesotheliomas [32]. Finally, CTLA-4 gene polymorphisms have been associated to increased susceptibility to multiple types of cancer such as breast [33], melanoma [34] gastric and colon cancers [35] and cervical carcinomas [36].

At present, the expression of CTLA-4 was only taken into account for the MG profiling indicating an association between different CTLA-4 single nucleotide variants and susceptibility to disease [37].

Thus, in this study, we assessed the expression of CTLA-4 both at mRNA and protein levels in fixed paraffin-embedded thymoma tissues. Furthermore, we also evaluated the correlation between CTLA-4 expression and clinico-pathologic characteristics and prognosis of patients with thymomas.

## RESULTS

### Study population

The complete list of patient characteristics is summarized in Table 1. Clinico-pathologic analyses according to WHO and Masaoka-Koga classifications are reported in Supplementary Tables 1 and 2, respectively. Extra-capsular invasive behavior was evidenced mainly in B2, B3 and C thymoma types (71, 83 and 100%, respectively) (Supplementary Table 1). In addition, concomitant MG is present in about 1/3 of thymoma patients, mainly in B2-B3 thymoma types (59 and 50%, respectively). In regard to extra-capsular invasion, it was more evident in IIB, III and IV Masaoka-Koga stages (Supplementary Table 2).

**Table 1:** Patient demographics and clinical features

	PATIENTS N=68 (100%)
**GENDER**	
** M**	33 (48.5%)
** F**	35 (51.5%)
**AGE (years)**	
** Range**	21-81
** 21-45**	14 (20.6%)
** 46-59**	19 (27.9%)
** >60**	35 (51.5%)
** Median**	62
**Myasthenia Gravis**	
** Yes**	22 (32.4%)
** No**	46 (67.6%)
**Tumor Histology**	
** A**	11 (16.2%)
** AB**	17 (25.0%)
** B1**	6 (8.8%)
** B2**	17 (25.0%)
** B3**	12 (17.6%)
** C**	5 (7.4%)
**TUMOR SIZE**	
** < 5 cm**	28 (41.2%)
** > 5 cm**	40 (58.8%)
**Invasion**	
** Yes**	55 (80.9%)
Capsular	24 (43.6%)
Extracapsular	31 (56.4%)
** No**	11 (16.2%)
** ND**	2 (2.9%)

**Table 2:** Univariate and multivariate analysis of overall survival in thymomas (Tumor CTLA-4 expression)

Variable	Univariate analyses			Multivariate analyses		
	HR	95% CI	P-value	HR	95% CI	P-value
-Tumor CTLA-4 expression	2.9987	1.4599	0.0107^*^	2.9301	1.2351	0.0235^*^
-Typical/Atypical	0.2170	0.029	0.0055^*^	0.1490	1.7970	0.0041^*^
-Myastenia Gravis	0.4874	0.2158	0.0889^ns^	0.6116	0.7171	0.1919^ns^
-Age	0.2145	0.0612	0.0002^*^	0.2971	1.6236	0.0071^*^
-Sex	1.4669	0.2516	0.4048^ns^	2.9641	0.4203	0.9807^ns^
-Tumor invasion	0.4150	0.6388	0.1745^ns^	0.4390	0.3312	0.5793^ns^
-Tumor dimension	0.4035	0.1634	0.0429^*^	0.3240	0.3814	0.6237^ns^
-Metastatic spread	1.4670	0.5050	0.5145^ns^	2.0072	0.4367	0.5149^ns^
-Radicality	1.7180	0.9976	0.0878^ns^	1.9703	0.9024	0.1531^ns^

Abbreviations: HR, hazard ratio; CI, confidence interval.^*^p < 0.05; ns = not significant.

### CTLA-4 mRNA expression in thymoma tissues

**Figure 1:**
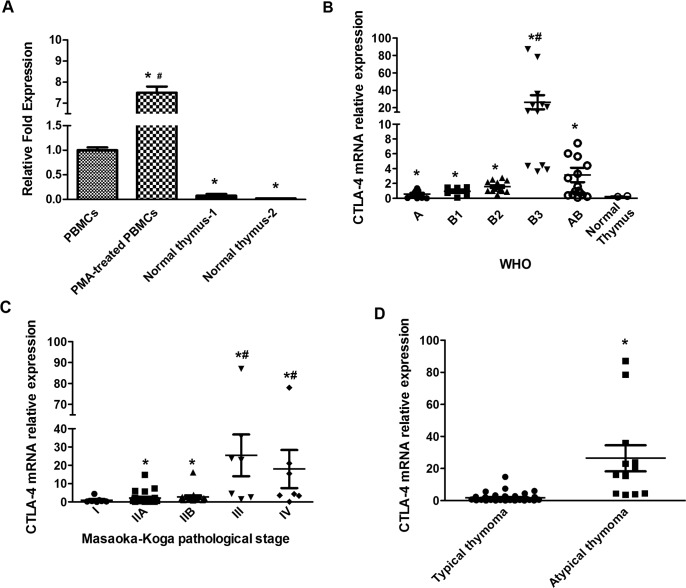
CTLA-4 expression on normal human thymus, lymphocytes and thymoma tissues **(A)** CTLA-4 mRNA expression was evaluated by qRT-PCR in normal human thymus and PBMCs treated or not with PMA. CTLA-4 mRNA levels were normalized for GAPDH expression. Data are expressed as fold mean ± SD. ^*^p<0.05 vs PBMCs, ^#^p<0.05 vs PBMC, normal thymus -1 and normal thymus -2. **(B)** CTLA-4 mRNA expression levels distribution according to WHO classification; statistical analysis was performed using non-parametric Kruskal-Wallis with Dunn's multiple comparisons test. Data are expressed as fold mean ± SD, ^*^p<0.05 vs normal thymus-2; ^#^p<0.05 vs A, B1, B2 and AB. **(C)** CTLA-4 mRNA expression levels distribution according to Masaoka-Koga classification; statistical analysis was performed by non-parametric Kruskal-Wallis with Dunn's multiple comparisons test. Data are expressed as fold mean ± SD, ^*^p<0.05 vs I, ^#^p<.0.05 vs IIA or IIB. **(D)** CTLA-4 mRNA expression level distribution according to Moran and Suster classification; statistical analysis was performed using non-parametric Kruskal-Wallis with Dunn's multiple comparisons test. Data are expressed as fold mean ± SD, ^*^p<0.05 typical vs atypical thymomas.

The CTLA-4 mRNA expression was evaluated in thymoma specimens (n = 63/68), two different batches of RNA from human normal thymus and, as control, peripheral blood mononuclear cells (PBMCs), from healthy donors, unstimulated and stimulated with phorbol 12-myristate 13-acetate (PMA). Given the small number of thymoma type C samples, the expression of CTLA-4 mRNA was not evaluated in these specimens. CTLA-4 mRNA was expressed at low levels in normal thymus, unstimulated PBMCs, whereas increased CTLA-4 levels (8-fold) were evidenced in PMA-stimulated PBMCs, upon PMA stimulation, as previously described [23, 38] (Figure 1A). Moreover, independently of WHO classification, CTLA-4 mRNA levels were very low in normal thymus respect to thymoma tissues (Figure 1B). Quantitative real time polymerase chain reaction (qRT-PCR) analysis also identified that the CTLA-4 mRNA expression progressively increase from A, B1, B2, AB and B3, with the higher levels in B3 type thymomas, as evaluated by statistical analysis (Figure 1B). Then, analyzing CTLA-4 expression in thymomas according to Masaoka-Koga stage, a significant difference between I vs IIA, IIB, III and IV, IIA and IIB vs III or IV stages was observed. No major differences were found comparing IIA vs IIB or III vs IV grade (Figure 1C). Finally, according to Moran and Suster classification [5, 7, 8, 10] significant difference in CTLA-4 mRNA expression were evidenced comparing typical (A, B1, B2 and AB types) vs atypical (B3 type) thymomas, with the last expressing very higher CTLA-4 mRNA levels respect to typical thymomas (Figure 1D). Finally, we evaluated whether adjuvant chemo-, radio-therapy or MG may affect the expression of CTLA-4 mRNA in thymoma patients (Supplementary Figure 1). We found higher CTLA-4 mRNA levels in thymoma patients undergoing adjuvant radiotherapy compared to untreated patients. No major differences in CTLA-4 expression were observed in untreated vs chemotherapy-administered thymoma patients and in MG vs non-MG thymoma patients.

### Expression of CTLA-4 protein in tumor cells and tumor-infiltrating leukocytes (TILs) from thymoma tissues

**Figure 2:**
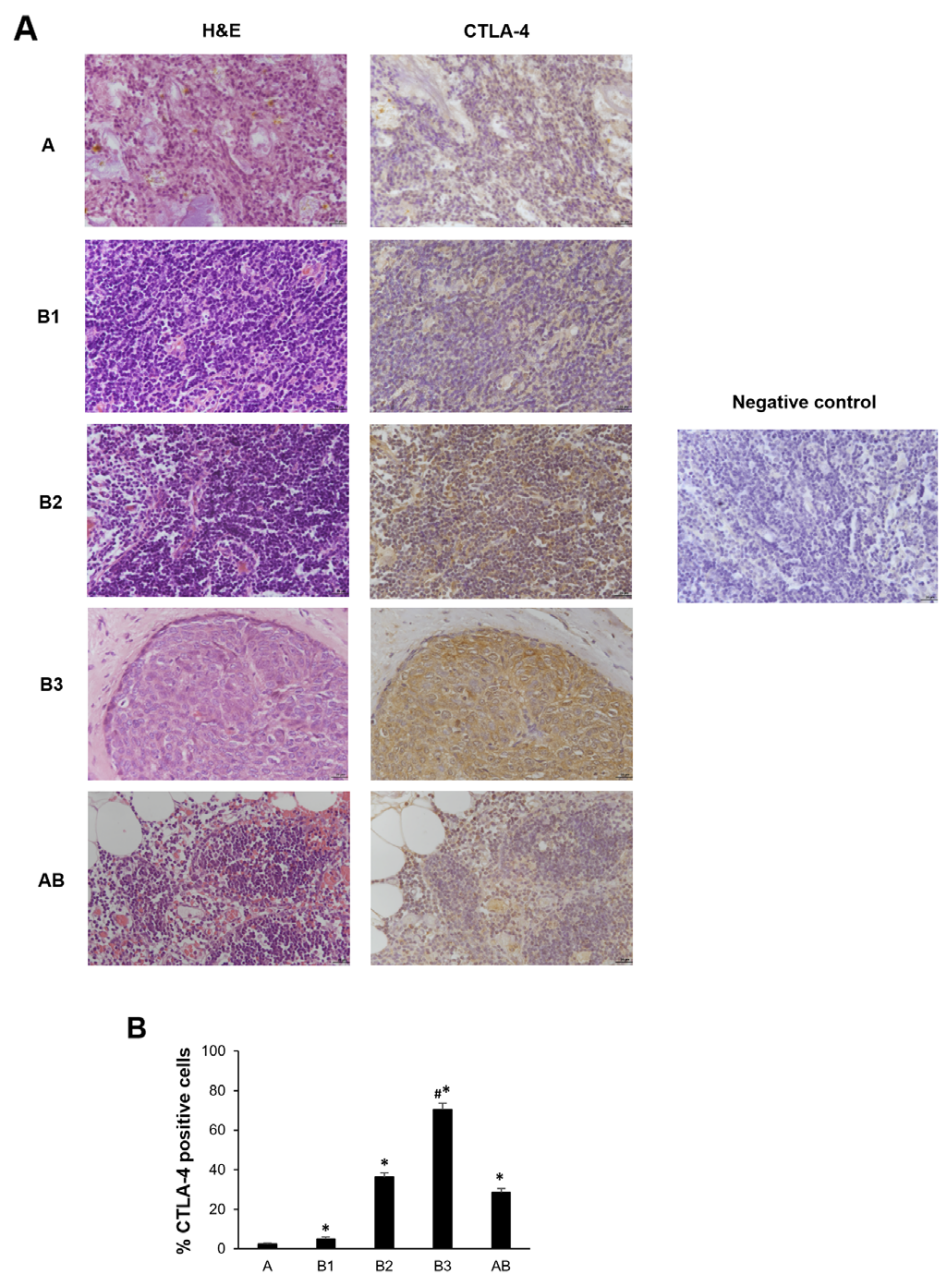
CTLA-4 protein expression in different subtypes of thymoma tissues **(A)** Sections of A, B1, B2, B3 and AB thymomas A were processed for hematoxylin and eosin staining (right column) and for CTLA-4 (left column) by immunohistochemistry. Sections of thymoma B1 thymomas processed for immunohistochemistry without incubation of primary antibody, were used as negative control. Calibration bar: 25 μm. **(B)** The percentage of CTLA-4 positively stained cells was determined in all samples according to the staining intensity by the NIS Software. Data represents the mean ± SD. ^*^p<0.05 vs A; ^#^p<0.05 vs B1, B2 and AB.

The results obtained in thymoma tissues by qRT-PCR prompted us to evaluate the expression of CTLA-4 protein in the different histological type of thymomas by immunohistochemistry (IHC) and semi-quantitative analysis. CTLA-4 immunoreactivity was found in all thymoma type sections and the percentage of CTLA-4^+^ cells progressively increase from A, B1, B2, AB and B3, with the highest levels in B3 type thymomas (Figure 2A and 2B). No reactivity was found in tissue sections used as negative control incubated with the omission of primary Ab (Figure 2A). Moreover, although the number of thymoma type C samples was limited to be included in the statistical analysis, the immunohistochemistry was performed and showed a strong CTLA-4 staining in these specimens (Supplementary Figure 2).

**Figure 3:**
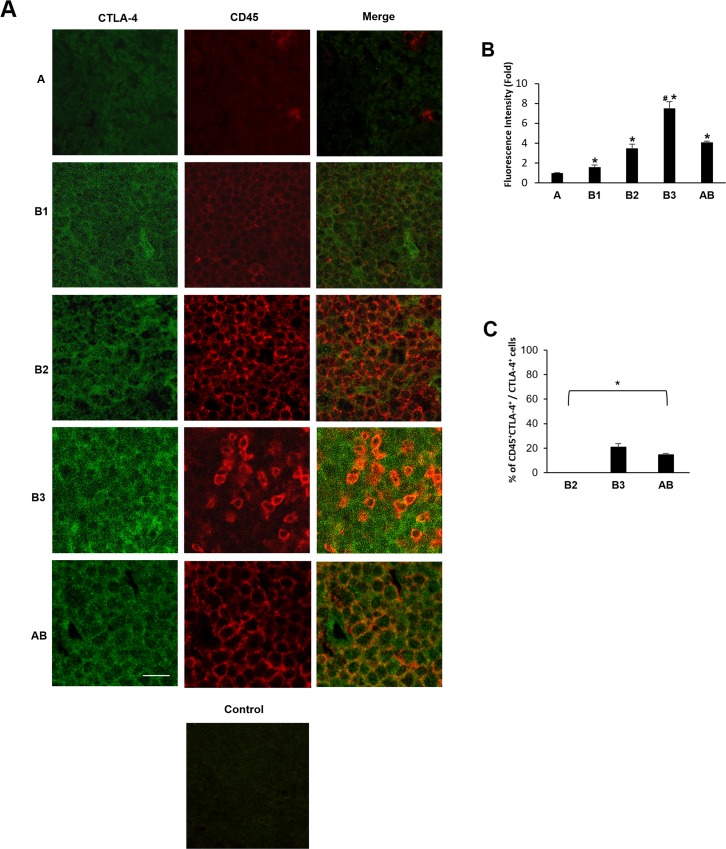
Expression of CTLA-4 in tumor cells and CD45^+^ TILs from thymoma specimens Confocal microscopy analysis was performed in thymoma tissues double-stained with anti-CTLA-4 and anti-CD45 mAbs, followed by Alexa Fluor 488- and Alexa Fluor 594-conjugated secondary Abs, respectively. Control = merge of secondary Abs without the incubation of the primary Abs. Data shown are representative of one out of three separate experiments. Calibration bar: 25 μm. **(B)** The CTLA-4 Fluorescence intensity was evaluated in all samples by using the NIS Software. Data represent the mean ± SD. ^*^p<0.05 vs **(A)**; ^#^p<0.05 vs B1, B2 and AB. **(C)** The percentage of CTLA-4^+^CD45^+^cells was evaluated in B2, B3 and AB samples according to the double fluorescence intensity by using the NIS Software and considering the total CTLA-4^+^ cells as 100%. Data represent the mean ± SD. ^*^p<0.05 vs total CTLA-4^+^ cells.

**Figure 4:**
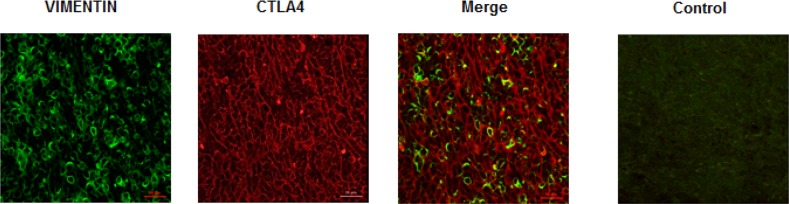
Expression of vimentin and CTLA-4 in AB subtype thymomas Confocal microscopy analysis was performed in thymoma AB specimens double-stained with anti-vimentin and anti-CTLA-4 mAbs, followed by Alexa Fluor 488- and Alexa Fluor 594-conjugated secondary Abs, respectively. Control = merge of secondary Abs without the incubation of the primary Abs. Data shown are representative of one out of three separate experiments. Calibration bar: 25 μm.

Since B1, B2 and B3 types are characterized by a proportional increase in TILs, the co-expression of CD45 and CTLA-4 antigens was evaluated using the anti-human CD45 and anti-CTLA-4 monoclonal Abs by confocal microscopy. CTLA-4 expression progressively increased in B1, B2, AB and B3 thymomas, whereas negligible CTLA-4 protein expression was found in type A thymoma as demonstrated by the Fluorescence Intensity confirming the IHC data (Figure 3A and 3B). Moreover, in tumor cells, CTLA-4 protein localised in the membrane, cytoplasm or both in a scattered pattern (Figure 3A). In addition, we found in accordance with previous reports [3, 4, 9], that TILs progressively increase from B1 to B3 and AB thymomas with about 20.1% and 14.6% of CTLA-4 positive cells being CD45 positive in B3 and AB thymomas respectively (Figure 3C). Finally, in AB thymomas the expression of vimentin and CTLA-4 in tumor cells was evaluated using an anti-human vimentin and anti-CTLA-4 mAbs. A mixture of less represented CTLA-4^+^/Vimentin^+^ tumor cells as well as a more represented CTLA-4^+^/Vimentin^−^ tumor cells was observed in AB thymoma sections (Figure 4).

### Correlation between clinico-pathologic and prognostic parameters, CTLA-4 tumor expression and overall survival in thymoma patients

**Figure 5:**
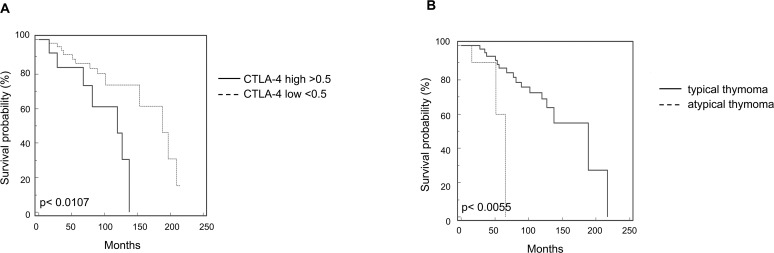
Kaplan-Meier curves of survival according to CTLA-4 expression and histological types **(A)** Kaplan-Meier plot was evaluated stratifying patients according to CTLA-4 expression levels. **(B)** Kaplan-Meier plot was evaluated stratifying patients according to typical vs atypical thymoma subtypes.

**Figure 6:**
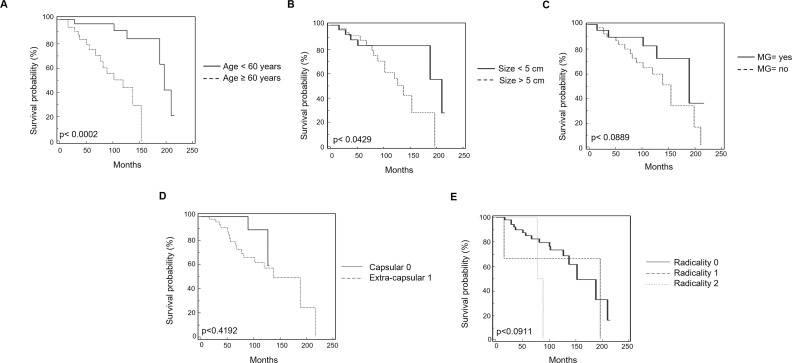
Kaplan-Meier curves of survival according to age, Myasthenia Gravis, tumor size, extra-capsular invasion and radicality **(A)** Kaplan-Meier plot was evaluated stratifying patients by age. **(B)** Kaplan-Meier plot was evaluated stratifying patients according to the tumor size. **(C)** Kaplan-Meier plot was evaluated stratifying patients according to the presence or not of Miastenia Gravis. **(D)** Kaplan-Meier plot was evaluated stratifying patients according to the presence of extra-capsular invasion or capsular invasion. **(E)** Kaplan-Meier plot was evaluated stratifying patients according to the radicality. R0: complete tumor resection; R1: incomplete microscopic tumor resection; R2: incomplete macroscopic tumor resection.

Survival curves were calculated according to univariate analysis (Table 2) and the Kaplan–Meier method by evaluating the age (≤ 60 vs > 60 years), sex, MG, invasion phenotype, invasion localization (capsular vs extra-capsular), tumor dimensions ≤ 5 cm or > 5 cm, radicality and CTLA-4 mRNA expression. In regard to CTLA-4 expression, patients were divided into two groups showing low < 0.5 (n = 15 specimens) and high > 0.5 (n = 48 specimens) CTLA-4 level. CTLA-4 mRNA expression reached significance for survival (p = 0.0107) (Figure 5A) with in CTLA-4^low^ OS = 188.31 vs CTLA-4^high^ OS = 119.50 months. In addition, atypical histological type thymoma (OS = 65.73 months) (Figure 5B) showed a significant (p = 0.0055) reduced survival respect to typical thymoma (OS = 188.22 months) confirming that high CTLA-4 expression in atypical thymoma (Figure 1D) is associated with negative prognosis. Age > 60 and tumor dimension > 5 cm were positively correlated (p = 0.0002, with OS ≤ 60 = 196.83 vs > 60 = 119.50 months; p = 0.0429, with OS ≤ 5cm = 209.88 vs > 5cm = 137.72 months, respectively) (Figure 6A and 6B). The presence of concomitant MG in thymoma patients approximates the significance (p = 0.0889, OS = 188.32 vs non MG = 153.66 months) (Figure 6C). No correlation for sex, tumor invasion, extra-capsular tumor invasion (Figure 6D) and radicality (Figure 6E) was evidenced. Thus, the higher CTLA-4 mRNA expression, age > 60 years and tumor dimension > 5 cm strongly correlated with short survival in total thymoma group and in atypical thymoma subgroup. Additionally we performed a multivariate analysis based on the Cox regression model to test the influence of CTLA-4 mRNA expression, age > 60 years and tumor dimensions > 5 cm on the survival of thymoma patients. We found that CTLA-4 overexpression, age > 60 years and typical vs atypical histological subtypes retained their prognostic negative significance in thymoma patients (p = 0.0235, 0.0071 and 0.0041, respectively) (Table 2).

## DISCUSSION

Thymic epithelial tumors (TETs) are uncommon neoplasms with a wide range of anatomical, clinical, histological and molecular malignant entities. At present, the management of TETs within clinical practice is based on a multimodal therapeutic strategy including surgery, chemotherapy and radiotherapy with a multidisciplinary approach and prognostic evaluation mainly based on Masaoka-Koga staging and WHO classification [39–41].

Novel strategies are needed, especially for refractory, recurrent thymic tumors after first-line chemotherapy failure. The investigation of molecular profiling and the analyses of immunological markers in thymic tumors could also allow determining potentially new targets.

To our knowledge, this is the first study demonstrating that elevated CTLA-4 tumor expression in thymoma patients correlates with poor prognosis and shorter OS. At present, CTLA-4 association to thymomas has only been taken into account for the MG profiling. In particular, single nucleotide polymorphisms (SNPs) of CTLA-4 seem to be associated with the manifestation of MG in patients with thymoma. SNPs in position 49 in patients expressing high CTLA-4 levels, in particular SNP +49 A/A and SNP +49 A/G showed a bad prognosis and reduced OS [42–44].

Herein, we analyzed the expression of CTLA-4 in thymoma patients with different WHO histological types and Masaoka-Koga stages. We found that CTLA-4 expression was significantly higher in all thymoma WHO types, compared with healthy thymus. We also stated that CTLA-4 gene expression was lower in A and B1, progressively increased in B2 and AB and was higher in B3 type thymomas, representing this histological type, the atypical thymoma subgroup, according to Moran and Suster classification [5, 7, 8, 10]. Thus, following both the WHO or Moran and Suster classifications the highest CTLA-4 value was evidenced in atypical B3 thymomas.

In parallel, by qRT-PCR, we observed a higher CTLA-4 expression in more advanced Masaoka-Koga grade IIB, III and IV compared to I and IIA, suggesting that CTLA-4 overexpression may be related to tumor aggressiveness. In regard to AB thymomas, a mixture of less represented mesenchymal CTLA-4^+^/Vimentin^+^ tumor cells as well as a more represented CTLA-4^+^/Vimentin^−^ tumor cells was observed in AB thymoma sections. The metaplastic CTLA-4^+^/Vimentin^+^ tumor cells likely derived from an epithelial mesenchymal transition process and trans-differentiation of spindle cells that have up-regulated mesenchymal markers [11].

According to previously reported data [12], Kaplan-Mayer and univariate analysis evidenced in thymoma patients with concomitant MG higher OS and good prognosis compared to non-MG patients. Autoimmunity and malignancy frequently coexist and they may share etiological and pathogenic mechanisms [45]. A sort of protective autoimmunity has been reported to improve the survival of cancer patients [46]; however at the other hand, better OS observed in MG patients could also depend to early discovery of MG vs non-MG thymomas, allowing a better survival of thymoma patients [47].

More importantly, our results suggest that in thymoma patients higher CTLA-4 mRNA expression represents a negative prognostic marker. In fact, the increase of CTLA-4 gene expression in total thymoma group and in B3 subgroup was associated with a shorter survival. In a multivariate Cox proportional hazards regression model, high CTLA-4 levels both in total and atypical thymoma, as well as age > 60 and tumor dimension > 5 cm, confirm their significance as negative prognostic factor for survival. Overall, tumor-associated CTLA-4 could promotes tumor progression by inhibiting the anti-tumor T cell immunity, inducing tumor-specific T cell apoptosis or impairing cytokine production and T cell-mediated cytotoxicity [48].

We also found that CTLA-4 is expressed not only in tumor cells, but also in CD45 leukocytes infiltrating the thymomas. In immune cells, CTLA-4 has been found to be expressed in B lymphocytes [17], monocytes [49], and activated effector CD4^+^ and CD8^+^ T cells, as well as constitutively expressed on a subset of regulatory Treg [50] participating in the co-stimulatory activation of naïve T cells or depletion of activated T cells [51]. Antigen specific activation of naïve T cells induces the expression of cytokines such as interferon-γ, which in turn triggers CTLA-4 expression in surrounding immune and tumor cells. The presence of Treg in tumor microenvironment has been associated with poor outcomes in patients with cancers [52]. In this regard, confocal microscopy analysis evidenced CTLA-4^+^CD45^+^ TILs, likely Treg lymphocytes, in the tumor microenvironment of B3 and AB thymomas, showing reduced OS. Thus the presence of CD45^+^CTLA-4^+^ TILs, in tumor environment of B3 and AB thymomas, might contribute to the profile of immunosuppression allowing unrestrained tumor progression due to impaired host immune surveillance.

The clinical implication of CTLA-4 expression in tumors or immune cells in the tumor microenvironment is still controversial, and the potential for CTLA-4 as prognostic and therapeutic marker has been complicated by differences in study population, methods and histological tumor types [53–54]. In agree with our results, univariate analysis demonstrates that higher tumor CTLA-4 expression is associated with shorter OS and poorer prognosis of patients with esophageal cancers [31]. Moreover in human breast cancer, high tumor CTLA-4 expression is associated with shorted OS, disease-free survival and worse prognosis [28]. The OS, failure-free survival and distant failure free survival rate was lower in nasopharyngeal carcinoma (NPC) patients and NPC patients with high tumor CTLA-4 levels [29]. By contrast, the CTLA-4 expression positively correlates with less advanced stage, intestinal type and well/moderately differentiated gastric adenocarcinoma [30] and represents a favorable prognostic factor in mesothelioma tissues, serum and pleural effusion [32]. Finally, a higher frequency of CTLA-4 overexpression was found in non-squamous as respect to squamous non-small cell lung cancer, and a reduced death rate was found in CTLA-4 overexpressing tumors [27].

Different cellular and murine models have been used to demonstrate that drugs inducing CTLA-4 blockade, used alone or in combination with other therapeutic interventions, improves endogenous responses to several tumor types, leading to tumor cell death when utilized. Treatment of CTLA-4 expressing tumor cell lines with recombinant forms of the CTLA-4 ligands CD80 and CD86 induced caspase-8-dependent apoptosis and the level of apoptotic tumor cells was reduced by soluble CTLA-4 and anti-CTLA-4 single-chain variable fragment antibodies [26]. Preclinical findings have been translated into the clinical development of two different CTLA-4 blocking Ab, ipilimumab (IPI) and tremelimumab [55–56]. Thus, antibody-mediated blockade of CTLA-4 may ultimately prove useful, either alone or in combination with other immune-based manipulations, have been successful employed in patients with metastatic melanoma, advanced prostate and pancreatic carcinomas [57–59], refractory B-cell non-Hodgkin lymphoma [60], early stage breast cancer [61] and we suggested it could be also used to improve the effectiveness of thymoma therapy. Moreover, in the view of increasing numbers of effective drugs available for treatment of thymoma, upfront identification of patients who are more likely to fail or benefit of treatment is the major unmet need. Regarding the use of IPI, thymoma patients showing high CTLA-4 expression could be benefit of immunotherapy with IPI. Thus, the higher CTLA-4 expression not only in Teff and Treg cells, but also in tumor cells, might facilitate tumor cellular lysis through an IPI-dependent cell-mediated cytoxicity by FcRγ expressing immune cells, such as monocytes for Treg [62] or natural killer and lymphocytes Tγδ for tumor cells [63]. In addition, CTLA-4 could also serve as a predictive biomarker for selecting the most appropriate therapy for thymoma patients and maximizing the clinical benefit with minimal toxicity. Other immunosuppressive co-stimulatory molecules, including PD-L1, PD-L2 remain under investigation in the context of thymomas and may similarly facilitate the downregulation of anti-tumor immune responses. In this regard, as demonstrated for PD-L1 immunotherapy, increased CTLA-4 expression where observed in patients undergoing adjuvant radiotherapy [64].

Overall, further clinical studies may be warranted to completely address the meaning of CTLA-4 expression in tumor cells and tumor-infiltrating leukocytes and/or of others co-stimulatory molecules to define the most effective treatment and prognosis in thymoma patients.

## MATERIALS AND METHODS

### Patients and thymoma specimens

The study population consisted of all consecutive patients aged 18 years or older (n = 68) who underwent surgery for thymomas from 1993 to 2013 at Università Politecnica delle Marche–Azienda Ospedaliero-Universitaria Ospedali Riuniti Umberto I – Lancisi – Salesi, Ancona, Italy. Other inclusion criteria included Eastern Cooperative Oncology Group performance status ≤ 2, adequate organ functions and no serious concomitant disease. All patients gave their consent and the local Ethical Committee approved the study (214439942015 approval number).

Paraffin-embedded tissues were prepared from surgically removed thymomas. Thick sections (5-7 μm) of thymoma tissues were collected and processed. Thymoma sections were stained with hematoxylin and eosin (H&E) and classified according to WHO histologic classification, in type A, AB, B1, B2, B3 and C, as well as Masaoka-Koga classification in I, IIA, IIB, III and IV stages.

### Stimulation of PBMCs with PMA

PBMCs were isolated from whole blood of healthy donors (ASUR 9, Macerata) by density gradient centrifugation on a Lympholyte solution (Cederlane, Burlington, Canada). After washing, purified cells were counted and then 10^7^ cells (1×10^6^/ml) were treated with 5 ng/ml of PMA (Sigma Aldrich, St Louis, MO, USA) for 48 h at 37°C, 5% CO_2_ and 95% of humidity in RPMI 1640 (Euroclone Ltd, Devon, UK) supplemented with 10% heat-inactivated fetal calf serum 2mM L-glutamine, 100 IU/ml of penicillin and 100 μg/ml of streptomycin (Euroclone Ltd). Untreated cells were used as control.

### Tissues, RNA extraction and reverse transcription

Total RNA from unstimulated and PMA-stimulated PBMCs was extracted by RNeasy Mini Kit (QIAGEN, Milan, Italy) whereas from fixed paraffin-embedded tissue slices by using “RNeasy® FFPE” kit (Qiagen). Two different lots of RNA from human normal thymus were purchased from Zyagen (San Diego, USA) and Clontech (Mountain Valley, CA). All RNA samples were eluted in the appropriate buffer and their concentration and purity evaluated by 260/280nm measurement. Five hundred nanograms of RNA were subjected to reverse transcription in a total volume of 25 μl using the high-capacity cDNA archive kit (PE Applied Biosystems, Foster City, CA, USA) according to the manufacturer's instructions. Five microliters of the resulting cDNA products were pre-amplified for 10 cycles using kit “RT^2^ PreAMP cDNA Synthesis” (QIAGEN). Two microliters of the resulting preAmp products was used as template for PCR quantification employing “RT^2^ qPCR Primer Assay” kit (QIAGEN).

### Quantitative real time polymerase chain reaction (qRT-PCR)

qRT-PCR was performed by using IQ5 Multicolor real-time PCR detection system (BioRad, Hercules, CA, USA). The reaction mixture contained the RT^2^ SYBR® Green qPCR Mastermix (QIAGEN), human CTLA-4 and GAPDH primers (RT^2^ qPCR Primer Assay for Human CTLA-4 and GAPDH, QIAGEN). The PCR parameters were 10 min at 95°C followed by 40 cycles of 95°C for 15 s and 60°C for 40 s. All samples were assayed in triplicate in the same plate. The relative amount of target mRNA was calculated by the 2^−ΔΔCt^ method, using GAPDH as a housekeeping gene.

### Immunohistochemistry (IHC)

For IHC, sections were pretreated in microwave for 10 min with Tris-HCl EDTA pH 9. After washes in PBS, sections were treated with 3% H_2_O_2_ for 20 min, washed, incubated for 1 h at room temperature with 3% bovine serum albumin and 0.3% Triton X-100 in PBS, and then overnight at 4°C with a mouse anti-CTLA-4 mAb (clone BNI3, isotype IgG2a, 1:100) (Novus Biologicals, Littleton, CO, USA). Thereafter, slides were incubated for 30 min at room temperature with a biotinylated secondary antibody, rinsed, and exposed for 30 min to the streptavidin–biotin complex (ABC Elite kit; Vinci Biochem, Vinci, Italy). Immunoreactivity was detected by the addition of diaminobenzidine (Vector, USA) for 5 min, counterstained with hematoxylin for 30 seconds and embedded in mounting medium. Four random fields of each tissue specimen were analyzed under 20X magnification using the Olympus BX51 Microscope and the Image J software (National Institutes of Health, Bethesda, MD, USA). The percentage of CTLA-4 positively stained cells was determined in all samples according to the staining intensity by the NIS Software (Nikon, Otawara, Japan). For each tumor specimens positive cells were counted in 10-fields of 0,5 mm^2^.

### Confocal microscope analysis

Sections were pre-treated in microwave for 10 min with Tris-HCl EDTA pH 9. After 2 washes in PBS sections were treated with 3% H_2_O_2_ for 20 min, washed, treated for 6 min with potassium permanganate solution (KMnO4 0.06%) and incubated for 1 h at room temperature with 3% bovine serum albumin and 0.3% Triton X-100 in PBS. After that sections were incubated overnight at 4°C with mouse anti-CTLA-4 mAb (Novus Biologicals, Littleton, USA, clone BNI3, isotype IgG2a, 1:25) followed by anti-mouse IgG AlexaFluor 488 secondary Ab (Thermo Fisher Scientific corporation, Waltham, USA, 1:100) for 60 min at 37°C, then overnight at 4°C with mouse anti-human CD45 mAb (Dako, Glostrup, Denmark, clone 2B11+PD7/26, isotype IgG1, 1:50) followed by anti-mouse IgG1 AlexaFluor 594 secondary Ab (Thermo Fisher Scientific corporation, 1:100) for 60 min at 37°C. In some experiments sections were incubated overnight at 4°C with mouse anti-CTLA-4 mAb (1:25) followed by anti-mouse IgG2a AlexaFluor 594 secondary Ab (Thermo Fisher Scientific corporation, 1:100) for 60 min at 37°C, then overnight at 4°C with mouse anti-human Vimentin mAb (Sigma, Saint Louis, USA, clone V9, isotype IgG1, 1:50) followed by anti-mouse IgG AlexaFluor 488 secondary Ab (Thermo Fisher Scientific corporation, 1:100) for 60 min at 37°C. Finally sections were embedded in mounting medium. Four random fields of each tissue specimen were analysed under 40X magnification using the Confocal Microscopy Nikon C2plus and the NIS software (Nikon, Otawara, Japan). The CTLA-4 Fluorescence Intensity was evaluated in all samples by using the NIS Software. For each tumor specimens fluorescence intensity was evaluated in 10-fields of 0,5 mm^2^. Moreover, to assess the contribution of CD45^+^ cells and tumor cells in the CTLA-4 expression, the percentage of CD45^+^CTLA-4^+^ was evaluated in B2, B3 and AB samples according to the Fluorescence intensity and considering the total CTLA-4^+^ cells as 100% by the NIS Software.

### Statistical analysis

Statistical analysis was perfomed using One Way-Anova and Two Way-Anova with Bonferroni's post-test. In addition, the non-parametric Kruskal-Wallis with Dunn's multiple comparisons was used to analyze CTLA-4 expression between the different WHO thymoma type histological and Masaoka-Koga stages.

The significant contribute of adjuvant chemo- or radiotherapy on the CTLA-4 mRNA expression in thymoma patients was evaluated by unpaired t' test, p < 0.05.

OS were estimated using Kaplan-Meier method with Rothman's 95% confidence intervals (CI) and compared across the groups using the log-rank test. We determined by Relative Operating Characteristic (ROC) curve analysis the CTLA-4 mRNA value that best discriminated between good and poor survival. Then, patients were divided for age, sex, invasiveness, extra-capsular or capsular invasion, tumor dimension more or less 5 cm, concomitant myasthenia gravis, WHO histological types and on the basis of CTLA-4 mRNA expression in CTLA-4^low^≤ 0.5 and High CTLA-4^high^ > 0.5 (evaluated by ROC analysis), and typical and atypical thymomas. Overall survival was defined as the interval between the date of surgery to death or last follow-up visit. These groups were subjected to univariate and multivariate survival analysis. For survival analysis, the Kaplan-Meier method was used. For univariate analysis of significance, the long-rank test or Cox analysis was used. The Cox proportional hazards model was used for multivariate analysis. p< 0.05 was considered as statistically significant. Statistical analysis was performed with MedCalc package (MedCalc® v16.4.3).

## SUPPLEMENTARY MATERIALS FIGURES AND TABLES



## Abbreviations

CTLA-4: Cytotoxic T lymphocyte antigen 4
OS: Overall Survival
MG: Myasthenia Gravis
Teff: effector T cells
Treg: regulatory T cells
PBMCs: Peripheral Blood Mononuclear Cells
PMA: Phorbol 12-Myristate 13-acetate
qRT-PCR: Quantitative real time polymerase chain reaction
TILs: Tumor-infiltrating Leukocytes
IHC: Immunohystochemistry
mAB: Monoclonal Antibody
TETs: Thymic Epithelial Tumors
SNPs: Single Nucleotide Polymorphisms
NPC: Nasopharyngeal Carcinoma
IPI: Ipilimumab
H&E: Hematoxylin Eosin

## References

[1] Den Bakker MA, Roden AC, Marx A, Marino M (2014). Histologic classification of thymoma: a practical guide for routine cases. J Thorac Oncol.

[2] Chen G, Marx A, Chen WH, Yong J, Puppe B, Stroebel P, Mueller-Hermelink HK (2002). New WHO histologic classification predicts prognosis of thymic epithelial tumors: a clinicopathologic study of 200 thymoma cases from China. Cancer.

[3] Chalabreysse L, Roy P, Cordier JF, Loire R, Gamondes JP, Thivolet-Bejui F (2002). Correlation of the WHO Schema for the Classification of Thymic Epithelial Neoplasms With Prognosis: A Retrospective Study of 90 Tumors. Am J Surg Pathol.

[4] Rieker RJ, Hoegel J, Morresi-Hauf A, Hofmann WJ, Blaeker H, Penzel R, Otto HF (2002). Histologic classification of thymic epithelial tumors: comparison of established classification schemes. Int J Cancer.

[5] Suster S, Moran CA (2005). Problem areas and inconsistencies in the WHO classification of thymoma. Semin Diagn Pathol.

[6] Detterbeck FC, Nicholson AG, Kondo K, Van Schil P, Moran C (2011). The Masaoka-Koga stage classification for thymic malignancies: clarification and definition of terms. J Thorac Oncol.

[7] Marchevsky AM, Gupta R, McKenna RJ, Wick M, Moran C, Zakowski MF, Suster S (2008). Evidence-based pathology and the pathologic evaluation of thymomas: the World Health Organization classification can be simplified into only 3 categories other than thymic carcinoma. Cancer.

[8] Moran CA, Suster S (2008). The World Health Organization (WHO) Histologic Classification of Thymomas: a reanalysis. Curr Treat Options Oncol.

[9] Roden AC, Yi ES, Jenkins SM, Donovan JL, Cassivi SD, Garces YI, Marks RS, Aubry MC (2015). Diagnostic significance of cell kinetic parameters in World Health Organization type A and B3 thymomas and thymic carcinomas. Hum Pathol.

[10] Moran CA, Weissferdt A, Kalhor N, Solis LM, Behrens C, Wistuba II, Suster S (2012). Thymomas I: a clinicopathologic correlation of 250 cases with emphasis on the World Health Organization schema. Am J Clin Pathol.

[11] Miki Y, Hamada K, Yoshino T, Miyatani K, Takahashi K (2014). Type AB thymoma is not a mixed tumor of type A and type B thymomas, but a distinct type of thymoma. Virchows Arch.

[12] Wilkins KB, Sheikh E, Green R, Patel M, George S, Takano M, Diener-West M, Welsh J, Howard H, Askin F, Bulkley GB (1999). Clinical and Pathologic Predictors of Survival in Patients with Thymoma. Ann Surg.

[13] Desmedt JE (1957). Nature of the defect of neuromuscular transmission in myasthenic patients: post-tetanic exhaustion. Nature.

[14] Engels EA (2010). Epidemiology of thymoma and associated malignancies. J Thorac Oncol.

[15] Salama AK, Hodi FS (2011). Cytotoxic T-lymphocyte-associated antigen-4. Clin Cancer Res.

[16] Magistrelli G, Jeannin P, Herbault N, Benoit De Coignac A, Gauchat JF, Bonnefoy JY, Delneste Y (1999). A soluble form of CTLA-4 generated by alternative splicing is expressed by nonstimulated human T cells. Eur J Immunol.

[17] Oaks MK, Hallett KM, Penwell RT, Stauber EC, Warren SJ, Tector AJ (2000). A native soluble form of CTLA-4. Cell Immunol.

[18] Sharpe AH, Freeman GJ (2002). The B7–CD28 superfamily. Nat Rev Immunol.

[19] Suwalska K, Pawlak E, Karabon L, Tomkiewicz A, Dobosz T, Urbaniak-Kujda D, Kuliczkowski K, Wolowiec D, Jedynak A, Frydecka I (2008). Association studies of CTLA-4, CD28, and ICOS gene polymorphisms with B-cell chronic lymphocytic leukemia in the Polish population. Hum Immunol.

[20] Monne M, Piras G, Palmas A, Arru L, Murineddu M, Latte G, Noli A, Gabbas A (2004). Cytotoxic T-lymphocyte antigen-4 (CTLA-4) gene polymorphism and susceptibility to non-Hodgkin's lymphoma. Am J Hematol.

[21] Kaufman KA, Bowen JA, Tsai AF, Bluestone JA, Hunt JS, Ober C (1999). The CTLA-4 gene is expressed in placental fibroblasts. Mol Hum Reprod.

[22] Nagaraju K, Raben N, Villalba ML, Danning C, Loeffler LA, Lee E, Tresser N, Abati A, Fetsch P, Plotz PH (1999). Costimulatory markers in muscle of patients with idiopathic inflammatory myopathies and in cultured muscle cells. Clin Immunol.

[23] Wang XB, Giscombe R, Yan Z, Heiden T, Xu D, Lefvert AK (2002). Expression of CTLA-4 by human monocytes. Scand J Immunol.

[24] Wang XB, Fan ZZ, Anton D, Vollenhoven AV, Ni ZH, Chen XF, Lefvert AK (2011). CTLA4 is expressed on mature dendritic cells derived from human monocytes and influences their maturation and antigen presentation. BMC Immunol.

[25] Pistillo MP, Tazzari PL, Palmisano GL, Pierri I, Bolognesi A, Ferlito F, Capanni P, Polito L, Ratta M, Pileri S, Piccioli M, Basso G, Rissotto L (2003). CTLA-4 is not restricted to the lymphoid cell lineage and can function as a target molecule for apoptosis induction of leukemic cells. Blood.

[26] Contardi E, Palmisano GL, Tazzari PL, Martelli AM, Falà F, Fabbi M, Kato T, Lucarelli E, Donati D, Polito L, Bolognesi A, Ricci F, Salvi S (2005). CTLA-4 is constitutively expressed on tumor cells and can trigger apoptosis upon ligand interaction. Int J Cancer.

[27] Salvi S, Fontana V, Boccardo S, Merlo DF, Margallo E, Laurent S, Morabito A, Rijavec E, Dal Bello MG, Mora M, Ratto GB, Grossi F, Truini M (2012). Evaluation of CTLA-4 expression and relevance as a novel prognostic factor in patients with non-small cell lung cancer. Cancer Immunol Immunother.

[28] Yu H, Yang J, Jiao S, Li Y, Zhang W, Wang J (2015). Cytotoxic T lymphocyte antigen 4 expression in human breast cancer: implications for prognosis. Cancer Immunol Immunother.

[29] Huang PY, Guo SS, Zhang Y, Lu JB, Chen QY, Tang LQ, Zhang L, Liu LT, Zhang L, Mai HQ (2016). Tumor CTLA-4 overexpression predicts poor survival in patients with nasopharyngeal carcinoma. Oncotarget.

[30] Kim JW, Nam KH, Ahn SH, Park DJ, Kim HH, Kim SH, Chang H, Lee JO, Kim YJ, Lee HS, Kim JH, Bang SM, Lee JS (2016). Prognostic implications of immunosuppressive protein expression in tumors as well as immune cell infiltration within the tumor microenvironment in gastric cancer. Gastric Cancer.

[31] Zhang XF, Pan K, Weng DS, Chen CL, Wang QJ, Zhao JJ, Pan QZ, Liu Q, Jiang SS, Li YQ, Zhang HX, Xia JC (2016). Cytotoxic T lymphocyte antigen-4 expression in esophageal carcinoma: implications for prognosis. Oncotarget.

[32] Roncella S, Laurent S, Fontana V, Ferro P, Franceschini MC, Salvi S, Varesano S, Boccardo S, Vigani A, Morabito A, Canessa PA, Giannoni U, Rosenberg I (2016). CTLA-4 in mesothelioma patients: tissue expression, body fluid levels and possible relevance as a prognostic factor. Cancer Immunol Immunother.

[33] Erfani N, Razmkhah M, Talei AR, Pezeshki AM, Doroudchi M, Monabati A, Ghaderi A (2006). Cytotoxic T lymphocyte antigen-4 promoter variants in breast cancer. Cancer Genet Cytogenet.

[34] Bouwhuis MG, Gast A, Figl A, Eggermont AM, Hemminki K, Schadendorf D, Kumar R (2010). Polymorphisms in the CD28/CTLA4/ICOS genes: role in malignant melanoma susceptibility and prognosis?. Cancer Immunol Immunother.

[35] Hadinia A, Hossieni SV, Erfani N, Saberi-Firozi M, Fattahi MJ, Ghaderi A (2007). CTLA-4 gene promoter and exon 1 polymorphisms in Iranian patients with gastric and colorectal cancers. J Gastroenterol Hepatol.

[36] Ivansson EL, Juko-Pecirep I, Gyllensten UB (2010). Interaction of immunological genes on chromosome 2q33 and IFNG in susceptibility to cervical cancer. Gynecol Oncol.

[37] Sun L, Meng Y, Xie Y, Zhang H, Zhang Z, Wang X, Jiang B, Li W, Li Y, Yang Z (2014). CTLA4 variants and haplotype contribute genetic susceptibility to myasthenia gravis in northern Chinese population. PLoS One.

[38] Cilio CM, Daws MR, Malashicheva A, Sentman CL, Holmberg D (1998). Cytotoxic T lymphocyte antigen 4 is induced in the thymus upon in vivo activation and its blockade prevents anti-CD3-mediated depletion of thymocytes. J Exp Med.

[39] Berardi R, De Lisa M, Pagliaretta S, Onofri A, Morgese F, Savini A, Ballatore Z, Caramanti M, Santoni M, Mazzanti P, Cascinu S (2014). Thymic neoplasms: an update on the use of chemotherapy and new targeted therapies. A literature review. Cancer Treat Rev.

[40] Berardi R, Morgese F, Garassino MC, Cascinu S (2015). New findings on thymic epithelial tumors: something is changing. World J Clin Oncol.

[41] Berardi R, De Lisa M, Pagliaretta S, Paolucci V, Morgese F, Savini A, Caramanti M, Ballatore Z, Onofri A, Cascinu S (2014). Thymic Malignancies in the Targeted Therapies Era. J Carcinog & Mutagen.

[42] Chuang WY, Ströbel P, Gold R, Nix W, Schalke B, Kiefer R, Opitz A, Klinker E, Müller-Hermelink HK, Marx A (2005). A CTLA4 high genotype is associated with myasthenia gravis in thymoma patients. Ann Neurol.

[43] Zheng K, Zhang J, Zhang P, Guo Y (2012). PTPN22 and CTLA-4 gene polymorphisms in resected thymomas and thymus for myasthenia gravis. Thorac Cancer.

[44] Wang XB, Kakoulidou M, Qiu Q, Giscombe R, Huang D, Pirskanen R, Lefvert AK (2002). CDS1 and promoter single nucleotide polymorphisms of the CTLA-4 gene in human myasthenia gravis. Genes Immun.

[45] Maverakis E, Goodarzi H, Wehrli LN, Ono Y, Garcia MS (2012). The etiology of paraneoplastic autoimmunity. Clin Rev Allergy Immunol.

[46] Toubi E, Shoenfeld Y (2007). Protective autoimmunity in cancer (review). Oncol Rep.

[47] Maggi G, Casadio C, Cavallo A, Cianci R, Molinatti M, Ruffini E (1991). Thymoma: results of 241 operated cases. Ann Thorac Surg.

[48] Laurent S, Queirolo P, Boero S, Salvi S, Piccioli P, Boccardo S, Minghelli S, Morabito A, Fontana V, Pietra G, Carrega P, Ferrari N, Tosetti F (2013). The engagement of CTLA-4 on primary melanoma cell lines induces antibody-dependent cellular cytotoxicity and TNF-α production. J Transl Med.

[49] Laurent S, Carrega P, Saverino D, Piccioli P, Camoriano M, Morabito A, Dozin B, Fontana V, Simone R, Mortara L, Mingari MC, Ferlazzo G, Pistillo MP (2010). CTLA-4 is expressed by human monocyte-derived dendritic cells and regulates their functions. Hum Immunol.

[50] Bergholdt R, Taxvig C, Eising S, Nerup J, Pociot F (2005). CBLB variants in type 1 diabetes and their genetic interaction with CTLA4. J Leukoc Biol.

[51] Ward FJ, Dahal LN, Wijesekera SK, Abdul-Jawad SK, Kaewarpai T, Xu H, Vickers MA, Barker RN (2013). The soluble isoform of CTLA-4 as a regulator of T-cell responses. Eur J Immunol.

[52] Callahan MK, Wolchok JD, Allison JP (2010). Anti-CTLA-4 antibody therapy: immune monitoring during clinical development of a novel immunotherapy. Semin Oncol.

[53] Hu P, Liu Q, Deng G, Zhang J, Liang N, Xie J, Zhang J (2017). The prognostic value of cytotoxic T-lymphocyte antigen 4 in cancers: a systematic review and meta-analysis. Sci Rep.

[54] Scorsetti M, Leo F, Trama A, D'Angelillo R, Serpico D, Macerelli M, Zucali P, Gatta G, Garassino MC (2016). Thymoma and thymic carcinomas. Crit Rev Oncol Hematol.

[55] Ribas A, Kefford R, Marshall MA, Punt CJ, Haanen JB, Marmol M, Hauschild A (2013). Phase III randomized clinical trial comparing tremelimumab with standard-of-care chemotherapy in patients with advanced melanoma. J Clin Oncol.

[56] Wolchok JD, Hodi FS, Weber JS, Allison JP, Urba WJ, Robert C (2013). Development of ipilimumab: a novel immunotherapeutic approach for the treatment of advanced melanoma. Ann NY Acad Sci.

[57] Hodi FS, O'Day SJ, McDermott DF, Weber RW, Sosman JA, Haanen JB, Gonzalez R, Robert C, Schadendorf D, Hassel JC, Akerley W, van den Eertwegh AJ, Lutzky J (2010). Improved survival with ipilimumab in patients with metastatic melanoma. N Engl J Med.

[58] Royal RE, Levy C, Turner K, Mathur A, Hughes M, Kammula US, Sherry RM, Topalian SL, Yang JC, Lowy I, Rosenberg SA (2010). Phase 2 trial of single agent Ipilimumab (anti-CTLA-4) for locally advanced or metastatic pancreatic adenocarcinoma. J Immunother.

[59] Slovin SF, Higano CS, Hamid O, Tejwani S, Harzstark A, Alumkal JJ, Scher HI, Chin K, Gagnier P, McHenry MB, Beer TM (2013). Ipilimumab alone or in combination with radiotherapy in metastatic castration-resistant prostate cancer: results from an open-label, multicenter phase I/II study. Ann Oncol.

[60] Ansell SM, Hurvitz SA, Koenig PA, LaPlant BR, Kabat BF, Fernando D, Habermann TM, Inwards DJ, Verma M, Yamada R, Erlichman C, Lowy I, Timmerman JM (2009). Phase I study of ipilimumab, an anti-CTLA-4 monoclonal antibody, in patients with relapsed and refractory B-cell non-Hodgkin lymphoma. Clin Cancer Res.

[61] McArthur HL, Diab A, Page DB, Yuan J, Solomon SB, Sacchini V, Comstock C, Durack JC, Maybody M, Sung J, Ginsberg A, Wong P, Barlas A (2016). A pilot study of preoperative single-dose ipilimumab and/or cryoablation in women with early-stage breast cancer with comprehensive immune profiling. Clin Cancer Res.

[62] Romano E, Kusio-Kobialka M, Foukas PG, Baumgaertner P, Meyer C, Ballabeni P, Michielin O, Weide B, Romero P, Speiser DE (2015). Ipilimumab-dependent cell-mediated cytotoxicity of regulatory T cells ex vivo by nonclassical monocytes in melanoma patients. Proc Natl Acad Sci U S A.

[63] Rajasekaran N, Chester C, Yonezawa A, Zhao X, Kohrt HE (2015). Enhancement of antibody-dependent cell mediated cytotoxicity: a new era in cancer treatment. Immunotargets and Therapy.

[64] Formenti SC, Demaria S (2013). Combining Radiotherapy and Cancer Immunotherapy: A Paradigm Shift. J Natl Cancer Inst.

