# Supplementary material for: High CTLA-4 expression correlates with poor prognosis in thymoma patients
Source: Oncotarget. 2018 Mar 30;9(24):16665–77. doi: 10.18632/oncotarget.24645 (PMC5908277; doi:10.18632/oncotarget.24645)
Supplement: Supplementary file 1 [file oncotarget-09-16665-s001.pdf]

High CTLA-4 expression correlates with poor prognosis in thymoma patients

SUPPLEMENTARY MATERIALS

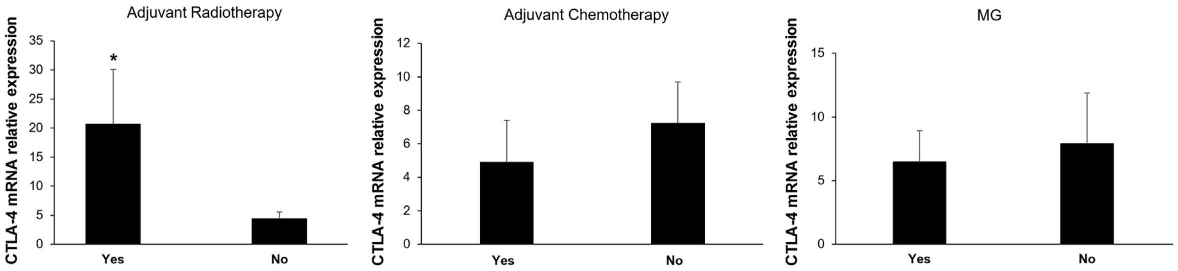

Supplementary Figure 1: CTLA-4 expression levels according to adjuvant radiotherapy, chemotherapy and Myasthenia Gravis. Patients were divided into two groups based on the presence (Yes) or absence (No) of adjuvant radio, chemotherapy or Miastenia Gravis. Data shown represent the mean of CTLA-4 mRNA expression levels (Fold) ± SEM evaluated by real time PCR. CTLA-4 mRNA levels were normalized for GAPDH expression. Statistical analysis was performed using unpaired *t*' test. *p<0.05 vs No radiotherapy.

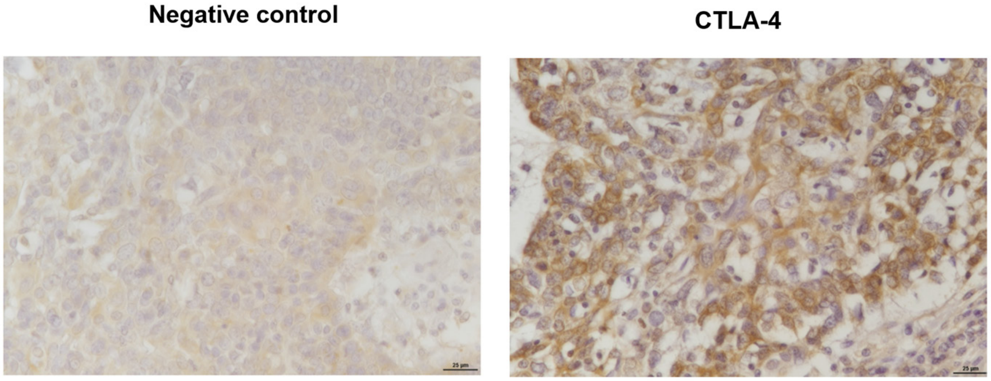

Supplementary Figure 2: Expression of CTLA-4 in C subtype thymomas. Sections of thymomas type C were processed for CTLA-4 by immunohistochemistry. Immunohistochemistry without the primary antibody was used as negative control. Data shown are representative of one out of five separate experiments. Calibration bar: 25 µm.

Supplementary Table 1: Clinico-pathologic analysis based on WHO classification

Characteristic	A	B1	B2	B3	AB	C	Total
**Sex**							
Male	5 (45.5)	6 (100.0)	5 (29.5)	5 (41.6)	9 (53.0)	3 (60)	33 (48.5)
Female	6 (54.5)	0 (0.0)	12 (70.5)	7 (58.4)	8 (47.0)	2 (40)	35 (51.5)
Total	11 (100)	6 (100)	17 (100)	12 (100)	17 (100)	5 (100)	68 (100)
**Age**							
<Median	1 (9.1)	3 (50.0)	11 (64.7)	8 (66.7)	8 (47.0)	4	35 (51.5)
≥Median	10 (90.9)	3 (50.0)	6 (35.3)	4 (33.3)	9 (53.0)	1	33 (48.5)
Total	11 (100)	6 (100)	17 (100)	12 (100)	17 (100)	5	68 (100)
**Invasion**							
No	1 (9.1)	1 (16.7)	2 (11.7)	0 (0.0)	7 (41.2)	0 (0)	11 (16.2)
Yes	10 (90.9)	5 (83.3)	14 (82.3)	12 (100)	9 (52.9)	5 (100)	55 (80.9)
Capsular	7 (70.0)	2 (40.0)	4 (28.6)	2 (16.7)	9 (100)	0 (0)	
Extra-capsular	3 (30.0)	3 (60.0)	10 (71.4)	10 (83.3)	0 (0)	5 (100)	
ND	0 (0)	0 (0)	1 (6.0)	0 (0)	1 (5.9)	0 (0)	2 (2.9)
Total	11 (100)	6 (100)	17 (100)	12 (100)	17 (100)	5	68 (100)
**Myasthenia Gravis**							
No	9 (81.8)	5 (83.4)	6 (35.3)	6 (50.0)	14 (82.4)	5 (100)	45 (66.2)
Yes	2 (18.2)	1 (16.6)	10 (58.8)	6 (50.0)	3 (17.6)	0 (0)	22 (32.3)
ND	0 (0)	0 (0)	1 (5.9)	0 (0)	0 (0)	0 (0)	1 (1.5)
Total	11 (100)	6 (100)	17 (100)	12 (100)	17 (100)	5	68 (100)
**Radicality**							
0	11 (100)	3 (60.0)	17 (100)	8 (72.7)	16 (100)	5	59 (86.8)
1	0 (0)	0 (0.0)	0 (0)	3 (27.3)	0 (0)		3 (4.4)
2	0 (0)	2 (40.0)	0 (0)	0	0 (0)		2 (2.9)
ND		1	1	1	1		4
Total	11 (100)	5 (100)	17 (100)	11 (100)	16 (100)	5	68 (100)

Radicality 0: complete tumor resection.
Radicality 1: incomplete microscopic tumor resection.
Radicality 2: incomplete macroscopic tumor resection.
ND: Not Determined.

Supplementary Table 2: Clinico-pathologic analysis based on Masaoka-Koga classification

Characteristic	I	IIA	IIB	III	IVA	IVB	Total
**Sex**							
Male	4 (57.1)	14 (52.0)	7 (39.0)	5 (71.4)	2 (33.3)	1 (33.3)	33 (48.5)
Female	3 (42.9)	13 (48.0)	11 (61.0)	2 (28.6)	4 (66.7)	2 (66.7)	35 (51.5)
Total	7 (100)	27 (100)	18 (100)	7 (100)	6 (100)	3 (100)	68 (100)
**Age**							
<Median	2 (28.6)	16 (57.1)	7 (38.9)	4 (66.7)	4 (57.1)	2 (66.7)	35 (51.5)
≥Median	5 (71.4)	12 (42.9)	11 (61.1)	3 (33.3)	1 (42.9)	1 (33.3)	33 (48.5)
Total	7 (100)	28 (100)	18 (100)	7 (100)	5 (100)	3 (100)	68 (100)
**Invasion**							
No	6 (85.7)	4 (14.3)	0 (0)	1 (14.3)	0 (0)	0 (0)	11 (16.2)
Yes	1 (14.3)	23 (82.1)	18 (100)	6 (85.7)	4 (80.0)	3 (100)	55 (80.9)
Capsular	1 (100)	21 (91.3)	2 (11.1)	0 (0)	0 (0)	0 (0)	
Extra-capsular	0 (0)	2 (8.7)	16 (88.9)	6 (100)	4 (100)	3 (100)	
ND	0 (0)	1 (3.6)	0 (0)	0 (0)	1 (20.0)	0 (0)	2 (2.9)
Total	7 (100)	28 (100)	18 (100)	7 (100)	5 (100)	3	68 (100)
**Myasthenia Gravis**							
No	6 (85.7)	20 (71.4)	10 (55.6)	5 (71.4)	2 (40.0)	2 (66.7)	45 (66.2)
Yes	1 (14.3)	7 (25.0)	8 (44.4)	2 (28.6)	3 (60.0)	1 (33.3)	22 (32.3)
ND	0 (0)	1 (3.6)	0 (0)	0 (0)	0 (0)	0 (0)	1 (1.5)
Total	7 (100)	28 (100)	18 (100)	7 (100)	5 (100)	3 (100)	68 (100)
**Radicality**							
0	7 (100)	28 (100.0)	16 (94.1)	3 (42.8)	4 (66.7)	1 (33.3)	59 (86.8)
1	0 (0)	0 (0)	1 (5.9)	2 (28.6)	0 (0)	0 (0)	3 (4.4)
2	0 (0)	0 (0)	0 (0)	2 (28.6)	0 (0)	0 (0)	2 (2.9)
ND	0 (0)	0 (0)	0 (0)	0 (0)	2 (33.3)	2 (66.7)	4 (5.9)
Total	7 (100)	28 (100)	17 (100)	7 (100)	6 (100)	3 (100)	68 (100)

Radicality 0: complete tumor resection.
Radicality 1: incomplete microscopic tumor resection.
Radicality 2: incomplete macroscopic tumor resection.
ND: Not Determined.
